# Practice of colorectal cancer screening in the United Arab Emirates and factors associated – a cross-sectional study

**DOI:** 10.1186/s12889-023-16951-7

**Published:** 2023-10-16

**Authors:** Latifa Nabeel Alsaad, Jayadevan Sreedharan

**Affiliations:** College of Medicine, Gulf Medical University, Ajman, UAE

**Keywords:** Colorectal cancer, CRC, CRC screening, CRC screening practice

## Abstract

**Background:**

Colorectal cancer is a significant public health concern globally, with high incidence and mortality rates. Despite the implementation of CRC screening guidelines, the uptake of screening among adults in the UAE remains low. This study aimed to assess the practice, factors associated, barriers, and knowledge gaps among adults in the UAE.

**Materials and methods:**

2100 residents of the UAE, aged > = 40 years, participated in the study. A validated questionnaire was used to collect data. Data was collected through online platforms and face-to-face interviews in healthcare settings. Chi-Square test and binary logistic regression were used for data analysis.

**Results:**

The study revealed a low CRC screening rate of 9.1%. Factors analyzed included age groups, health insurance coverage, regular physician checkups, family history of CRC, awareness of CRC, and knowledge levels about CRC and its signs and symptoms. Participants in the 50–59 age group showed a slightly higher likelihood of CRC screening, but the difference was not statistically significant. However, individuals in the 60–69 and > = 70 age groups were more likely to undergo screening. Regular physician checkups, family history of CRC, prior knowledge of CRC, and knowledge about the disease and its signs and symptoms were associated with a higher likelihood of screening, with statistically significant OR.

**Conclusion:**

A low CRC screening rate of 9.1% among adults. Barriers to screening included not being offered a test by physicians, fear of positive results, discomfort with the screening process, perception of pain, and lack of knowledge. Identifying particulate barriers and developing targeted measures requires larger-scale research.

## Introduction

Colorectal cancer (CRC) poses a serious public health threat due to its high incidence, prevalence, and mortality rates. The International Agency for Research on Cancer (IARC) estimates that CRC accounted for approximately two million cases and one million deaths globally in 2020, making it the third most prevalent cancer and the second leading cause of cancer-related deaths [2].

In the United Arab Emirates (UAE), colon cancer is the most common primary cancer among men and the third most common cancer in women, following breast and thyroid cancer, as per the 2019 annual report of the National Cancer Registry of the Ministry of Health and Prevention (MOHAP) [3]. Recognizing the importance of early detection, the UAE introduced colorectal cancer screening in 2013 [4]. Guidelines recommend regular colonoscopies or fecal immunochemical tests (FIT) for individuals aged 40 and above, with more frequent screenings for those at higher risk [5].

However, despite the availability of screening programs, the uptake of colorectal cancer screening among Emirati adults remains low. A study concluded that 90% of CRC cases were diagnosed early and effectively treated, but, according to the Health Authority of Abu Dhabi (HAAD), 63% of cases usually present late [6, 7]. This research aims to explore the factors influencing the utilization and non-utilization of colorectal cancer screening programs among adults in the UAE, shedding light on both facilitating and hindering factors.

While research on colorectal cancer is evolving globally, there is limited published data specific to the UAE context. Therefore, this study’s findings aim to contribute to the development of targeted public health interventions aimed at improving knowledge and practices related to CRC screening among adults in the UAE. By identifying barriers and facilitators, this research will assist in addressing the CRC epidemic and promoting the uptake of screening tests.

## Materials and methods

This study is a cross-sectional study conducted over a period of 15 weeks in the months of March to June 2023. The study aimed to investigate the practice of colorectal cancer screening among residents of the United Arab Emirates aged 40 years and above. The study included participants of different nationalities residing in all seven emirates of the UAE, provided they could read and understand Arabic or English and gave their informed consent to participate.

The sample size formula for cross-sectional studies with binary exposure was used to calculate the sample size based on the information from Al Abdouli et al. [3]. The practice of CRC screening observed in the study was 5%. Hence, P = 0.05 and q = 0.95. The error is taken as 20% of the 0.05, which is 0.01. Thus, the minimum required sample size observed was 1900. Expecting a non-response rate of 10% and hence increasing the size by 20% of the calculated sample size. Thus, the final sample size was approximated to be 2100.

The researchers recruited participants through social media platforms and from various hospitals and health centers across the country after obtaining approvals from Gulf Medical University (approval number: IRB/COM/STD/80/DEC-2022) and the Ministry of Health and Prevention in the UAE (approval number: MOHAP/DXB-REC/F.M.M /No.19 /2023).

To gather data, a questionnaire was developed based on the data available in the literature [3, 7, 14]. The draft questionnaire was sent to three experts for validation. Among those experts, two were medical doctors with public health experience, and the third was an epidemiologist and statistician. The major domains of the questionnaire were sociodemographic details, health-related details, knowledge of colorectal cancer and screening, and practice of colorectal cancer screening. With a total length of three pages, the questions were offered mainly as closed-ended questions, with a few open-ended questions. An English version of the questionnaire was developed first, which was then translated into Arabic to ensure the elimination of language barriers. Back translation to English was also performed before collecting the data to ensure that the content of both versions remained consistent and accurate. A pilot study was done prior to recruiting the participants in this study. The aim was to assess the clarity and acceptability of the questions from the participant’s point of view, the face validity of the questionnaire, the response variability, and the time required for the participants to complete the survey. In the pilot study, participants were recruited using both English and Arabic versions of the questionnaire. Based on the responses received from participants, the questionnaire was then updated to its final version and was ready to be used in this cross-sectional study. Ethical considerations were taken seriously throughout the study, with participants required to provide informed consent. Anonymity, privacy, and confidentiality were maintained, and participants were assured that their information would be used for research purposes only.

Data collection occurred both online and face-to-face, with the completeness of the questionnaire checked before inclusion in the final analysis. The collected data were stored at Gulf Medical University, following accepted ethical practices.

Data analysis was performed using SPSS software version 28. Descriptive statistics were used to determine the practice of colorectal cancer screening. The Chi-Square test was employed to identify factors associated with the practice of colorectal cancer screening, and both simple and multiple binary logistic regressions were used to determine factors associated with the non-utilization of the screening program.

## Results

In this study, 2100 individuals participated. Among them, 51.9% (n = 1090/2100) were males, 53.9% (n = 1131/2100) were in the age group of 40–49 years, 81.8% (n = 1717/2100) were from the Eastern Mediterranean Region (EMR), and 65.6% (n = 1378/2100) had a tertiary level of education. The vast majority of the respondents (80.3%, n = 1686/2100) were married. The details are given in Table 1.

**Table 1:** Sociodemographic Characteristics of the Participants

Sociodemographic Variables	Group	Frequency	%
Gender	Male	1090	51.9
	Female	1010	48.1
Age	40–49	1131	53.9
	50–59	631	30.0
	60–69	291	13.9
	>= 70	47	2.2
Nationality	AFR*	74	3.5
	EMR*	1717	81.8
	EUR, AMR and WPR*	77	3.7
	SEAR*	231	11.0
Place of residence	Abu Dhabi	244	11.6
	Dubai	635	30.2
	Sharjah	375	17.9
	Ajman	475	22.6
	Fujairah	125	6.0
	Umm al Quwain	101	4.8
	Ras Al Khaimah	145	6.9
Marital status	Unmarried	197	9.4
	Married	1686	80.3
	Widow	113	5.4
	Divorced	104	5.0
Education level	*Primary	88	4.2
	Secondary (7–12)	634	30.2
	University or higher	1378	65.6

*AFR -African Region, EMR -Eastern Mediterranean Region, EUR -European Region, AMR -Region of the Americas, WPR -Western Pacific Region, SEAR -South-East Asian Region

** No formal education to grade 6

**Fig. 1:**
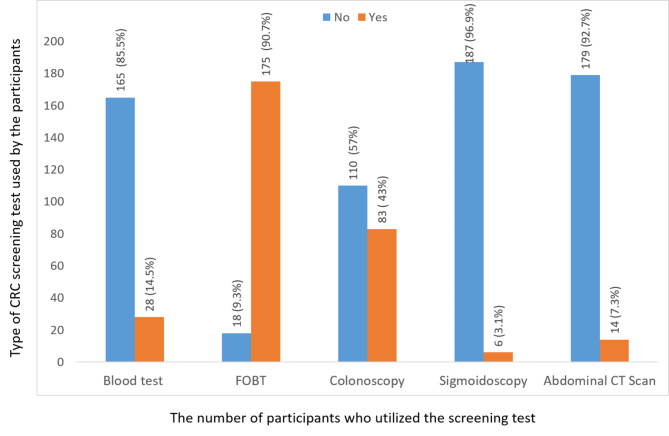
Screening methods utilized for colorectal cancer screening by the participants

Among all participants, 9.1% (n = 192/2100) screened for CRC. Their reasons for screening were either they were advised by their physician (4.6%, n = 97/192), just for regular screening (3.8%, n = 80/192), requested to be screened because of family history (0.6%, n = 13/192), screened because they were inflammatory bowel disease (IBS) patients (0%, n = 1/192), and other reasons (0.1%, n = 2/192). Figure 1 demonstrates the screening methods utilized for CRC screening by the participants.

### Factors hindering the practice of colorectal cancer screening

The participants were given a list of factors that could hinder their decision to undergo CRC screening. They were asked to indicate whether each factor would be a hindrance by selecting “yes” or “no”.

**Fig. 2:**
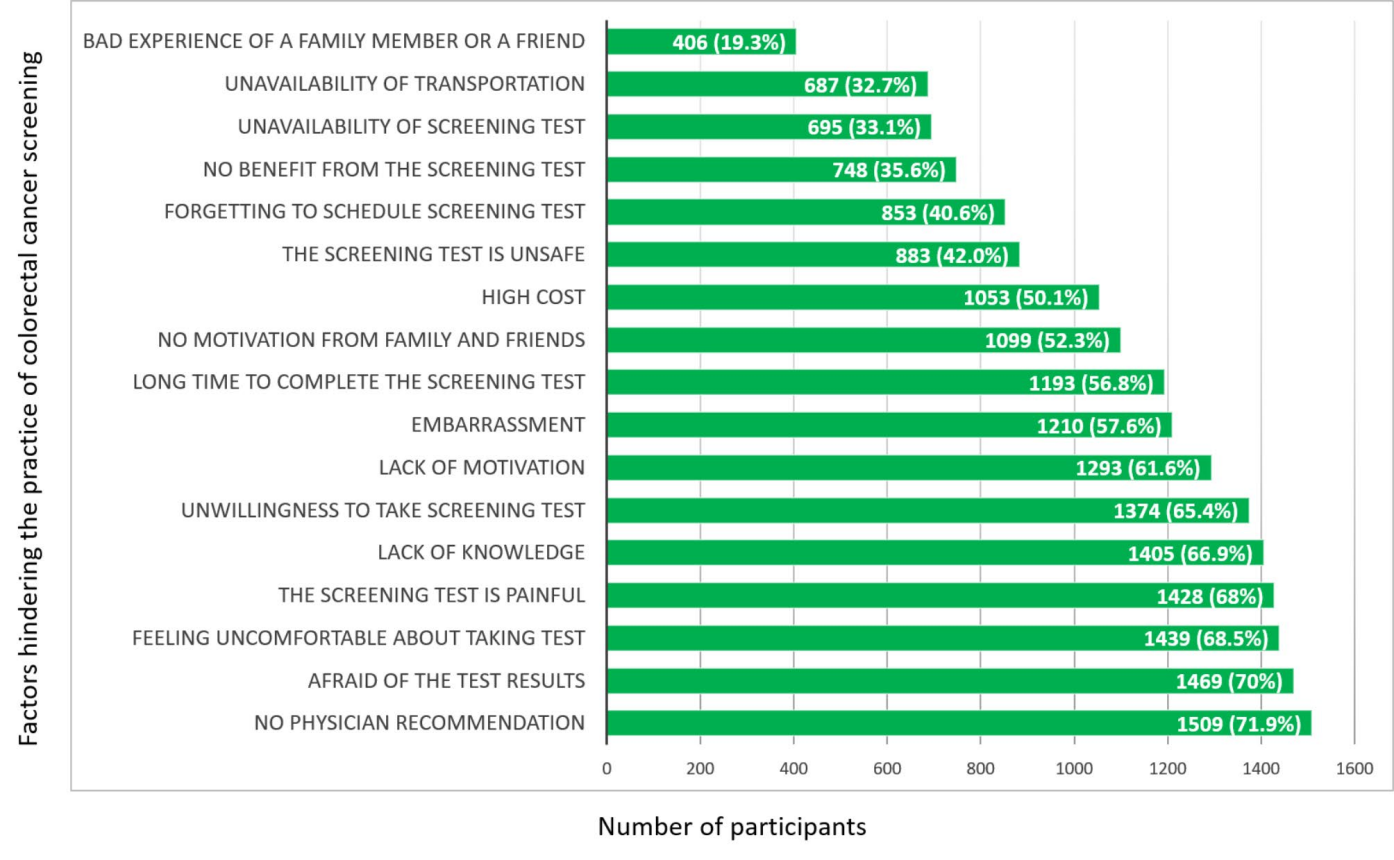
Factors hindering the practice of colorectal cancer screening according to the perception of the participants

Among the contributors in this study, 71.9% (n = 1509/2100) considered not being offered a CRC test by their physician to be a hindering factor in practicing screening. While 70% (n = 1469/2100) identified fear of test results as a factor hindering them from getting screened for CRC, 42% (n = 883/2100) thought the screening test was unsafe, and 68% (n = 1482/2100) thought it was painful. It was found that 68.5% (n = 1439/2100) did not feel comfortable about taking the test. Among the recruited participants, 50.1% (n = 1053/2100) found the cost of screening tests to be high, thus making it difficult for them to get screened. A lack of knowledge was also considered an obstacle to CRC screening by around two-thirds of the participants (66.9%, n = 1405/2100). Other factors were also identified by the participants that would hinder them from going for CRC screening. The details of all factors are given in Fig. 2.

### Association of practice of Colorectal cancer screening and sociodemographic variables

Table 2 demonstrates the association of the CRC screening practice based on the participants’ sociodemographic characteristics. 19.1%, >=70 years screened for CRC, whereas 15.8%, 10%, and 6.5%, respectively, for the age groups 60–69, 50–59, and 40–49. As age increases, the chance for CRC screening increases (P < 0.001). Regarding gender, 9.4% of males and 8.8% of females were screened for CRC. The association was not statistically significant. No statistically significant association was observed between education level and the practice of CRC screening. 11.1% of those with insurance and 13.3% of those without insurance were screened for CRC. There was an association between health insurance and the practice of CRC screening (P < 0.001).

**Table 2:** Association of Practice of Colorectal Cancer Screening vs. Sociodemographic Variables

Variable	Group	Practice of CRC Screening				Total	P- value
		No		Yes			
		No.	%	No.	%		
Age	40–49	1057	93.5	74	6.5	1131	< 0.001
	50–59	568	90.0	63	10.0	631	
	60–69	245	84.2	46	15.8	291	
	>= 70	38	80.9	9	19.1	47	
Gender	Male	987	90.6	103	9.4	1090	NS
	Female	921	91.2	89	8.8	1010	
Educational level	No formal education to grade 6	77	87.5	11	12.5	88	NS
	Secondary (7–12)	568	89.6	66	10.4	634	
	University or higher	1263	91.7	115	8.3	1378	
Nationality	AFR	69	93.2	5	6.8	74	NS
	EMR	1560	90.9	157	9.1	1717	
	EUR/ AMR/WPR	69	89.6	8	10.4	77	
	SEAR	209	90.5	22	9.5	231	
Marital status	Unmarried	189	95.9	8	4.1	197	< 0.01
	Married	1719	90.3	184	9.7	1903	
Health insurance	No	755	94.0	48	6.0	803	< 0.001
	Yes	1153	88.9	144	11.1	1297	

NS-Not statistically significant

### Association of practice of colorectal cancer screening and health-related factors

Table 3 illustrates the association between the CRC screening practice and the participants’ health-related factors. 13.3% of those with regular physician checkups and 5.6% of those without regular physician checkups practiced CRC screening. The association observed was statistically significant (P < 0.001). A family history of CRC showed a statistically significant association with CRC screening (P < 0.001). 27.1% of participants had a family history of CRC, and 7.6% of those with no family history of CRC were screened for CRC. There were more chances of CRC screening practice for those with a positive family history of CRC. Of those who heard about CRC, 10.3%, and those who did not hear, 2.7% screened for CRC, which showed the knowledge is leading to the practice. The association observed was statistically significant (P < 0.001). The association between the knowledge about the presence of CRC as a disease and the practice of CRC screening is statistically significant (< 0.001). Lastly, the association between the knowledge of screening benefits to provide timely and effective treatment for CRC and the CRC screening practice is not statistically significant.

**Table 3:** Association of Practice of Colorectal Cancer Screening vs. Health-Related Factors

Variable	Group	Practice of CRC Screening				Total	P- value
		No		Yes			
		No.	%	No.	%		
Health insurance	No	755	94.0	48	6.0	803	< 0.001
	Yes	1153	88.9	144	11.1	1297	
Regular physician checkup	No	1068	94.4	63	5.6	1131	< 0.001
	Yes	840	86.7	129	13.3	969	
Family history of CRC	No	1787	92.4	147	7.6	1934	< 0.001
	Yes	121	72.9	45	27.1	166	
Have ever heard of CRC	No	320	97.3	9	2.7	329	< 0.001
	Yes	1588	89.7	183	10.3	1771	
Screening help treat CRC	No	462	93.0	35	7.0	497	*NS
	Yes	1446	90.2	157	9.8	1603	

*Not significant

### Association of practice of colorectal cancer screening and knowledge about CRC

The knowledge variables were merged to assess the association between knowledge about CRC disease and the practice of CRC screening. The seven questions that were included to assess the knowledge of CRC awareness are: Is colorectal cancer a deadly disease? Is there a screening for colorectal cancer? Can colorectal cancer be prevented? Is there a treatment for colorectal cancer? Is colorectal cancer a curable disease? Can screening help in providing timely and effective treatment for colorectal cancer? And what is the appropriate age group for colorectal cancer screening in the UAE? In the assessments, a correct answer will be scored as one, and a wrong answer will receive a score of zero, for a total score of seven for the knowledge of CRC as a disease. A score of < 4 was considered below average level of knowledge, and > = 4 was considered above average. Among participants with a below-average level of knowledge, 4.3%, and an above-average level of knowledge, 12.8% were screened for CRC. The association observed was statistically significant (P < 0.001). All the surveyed colorectal cancer signs and symptoms were merged into one domain to study the association with CRC screening. These signs and symptoms are anemia, dark-colored stool, abdominal pain or cramps, diarrhea, constipation, feeling of incomplete bowel emptying, unintentional weight loss, unexplained weakness or tiredness, and loss of appetite. A score of < 5 was considered below average level of knowledge, and > = 5 was considered above average. Among the participants, the level of knowledge of CRC signs and symptoms was below-average in 4.2% and above-average in 10.9% among those who got screened for CRC. The association was statistically significant (P < 0.001). The details are given in Table 4.

**Table 4:** Association of Practice of Colorectal Screening Vs. Knowledge about CRC

Variable	Group	Practice of CRC Screening				Total	P- value
		No		Yes			
		No.	%	No.	%		
Knowledge about CRC disease	Below Average knowledge	864	95.7	39	4.3	903	< 0.001
	Above Average knowledge	1044	87.2	153	12.8	1197	
Knowledge about CRC signs and symptoms	Below Average knowledge	521	95.8	23	4.2	544	< 0.001
	Above Average knowledge	1387	89.1	169	10.9	1556	

### The crude odds ratio of the practice of CRC screening and sociodemographic variables

There was a trend of statistically significant increasing odds ratio as age increased (P < 0.01). It was observed that the odds of those who are > = 70 years old are more likely to practice CRC screening by 3.38 times. Compared to the participants who did not have health insurance, those covered by health insurance were 1.96 times more likely to take the CRC screening test (P < 0.001). The practice of CRC screening among those who attended regular physician checkups was 2.6 times higher based on the simple binary logistic regression (P < 0.001). A positive family history of CRC increased the likelihood of practicing CRC screening among the participants by 4.52 (P < 0.001). Participants who had ever heard of colorectal cancer were 4.10 times more likely to practice CRC screening (P < 0.001). The respondents who had an above-average level of knowledge were 3.3 times more likely to practice CRC screening, and based on their knowledge about CRC signs and symptoms, the participants who had an above-average level of knowledge were 2.8 times more likely to practice CRC screening compared to those who had a below-average level of knowledge. The OR was found to be statistically significant (P < 0.001). The details are given in Table 5.

### The adjusted odds ratio of the practice of CRC screening and sociodemographic variables

Multiple logistic regression was used to calculate the adjusted OR. Participants in the 50–59 age group were 1.56 times more likely (P < 0.05) to get screened for CRC, those who are in their 60–69 years and those who are > = 70 years are more likely to practice CRC screening by 2.97 (P < 0.001) and 3.18 times (P < 0.01) respectively, compared to the participants who are in their 40–49 years. It was also found that among the participants, compared to the unmarried, married participants were 1.85 times more likely to go for the CRC screening; the observed odds ratio was not statistically significant. Compared to the participants who did not have health insurance, those covered by health insurance were 1.22 times more likely to take the CRC screening test, but the observed adjusted OR was not statistically significant. The adjusted OR, 1.88, was statistically significant (P < 0.001) for CRC screening among those who attended regular physician checkups. Those who went to their physicians for regular checkups were more likely to practice CRC screening than those who did not. Family history of CRC increased the likelihood of practicing CRC screening among the participants by 3.21 times compared to those with a negative family history of the disease (P < 0.001) after adjusting with other variables. Participants who had ever heard of colorectal cancer were 2.1 times more likely to practice CRC screening (P < 0.05). Those with above-average levels of knowledge were 2.59 times more likely to practice CRC screening compared to those who were at a knowledge level that was below average (P < 0.001). Also, based on their knowledge of CRC signs and symptoms, the participants who had an above-average level of knowledge were 1.8 times more likely to practice CRC screening compared to those who had a below-average level of knowledge (P < 0.01).

**Table 5:** Crude and Adjusted Odds Ratio (OR) of Colorectal Cancer (CRC) Screening Practice

Variable	Group	Crude			Adjusted		
		OR	CI	P	OR	CI	P
Age	40–49	1			1		
	50–59	1.58	1.12–2.25	< 0.01	1.56	1.09–2.25	< 0.05
	60–69	2.68	1.81–3.97	< 0.001	2.97	1.96–4.50	< 0.001
	>=70	3.38	1.58–1.26	< 0.01	3.18	1.41–7.17	< 0.01
Marital status	Unmarried	1			1		
	Married	2.53	1.23–5.21	< 0.01	1.85	0.88–3.88	NS
Health insurance	No	1			1		
	Yes	1.96	1.40–2.76	< 0.001	1.22	0.83–1.79	NS
Regular physician check-up	No	1			1		
	Yes	2.60	1.90–3.57	< 0.001	1.88	1.32–2.68	< 0.001
Family history of CRC	No	1			1		
	Yes	4.52	3.09–6.62	< 0.001	3.21	2.15–4.80	< 0.001
Have ever heard of CRC	No	1			1		
	Yes	4.10	2.08–8.09	< 0.001	2.10	1.04–4.24	< 0.05
Knowledge about CRC disease	Below Average knowledge	1			1		
	Above Average knowledge	3.25	2.26–4.67	< 0.001	2.59	1.77–3.80	< 0.001
Knowledge about CRC signs and symptoms	Below Average knowledge	1			1		
	Above Average knowledge	2.8	1.77–4.32	< 0.001	1.80	1.13–2.85	< 0.01

## Discussion

This study identified the practice of colorectal cancer screening programs and the factors associated among adults aged > = 40 years who are residing in the United Arab Emirates.

### The practice of colorectal cancer screening

Our study found the practice of CRC screening to be 9.1%. This is higher than what was found in Saudi Arabia [8]. On the other hand, higher screening practices were found in Spain and the USA, with 38% and 80%, respectively [9, 10] The vast difference in the rate of screening practice could be due to differences in healthcare policies influencing guidelines between these countries. National policies and guidelines can influence screening practices in the sense that countries that have implemented systematic screening programs and have policies supporting routine screening tend to have higher screening rates for CRC. Not to mention, the differences in the resources available for screening differ between regions of the world. Cultural attitudes and beliefs can influence the uptake of colorectal cancer screening, based on studies done on Americans from different cultural backgrounds [11, 12]. Sociocultural factors, including modesty or the presence of stigma surrounding specific screening procedures, can influence individuals’ reluctance to undergo colorectal cancer screening. This finding aligns with the responses of over half of the participants in our study, who cited embarrassment and discomfort as reasons for not undergoing the test.

### Factors hindering colorectal cancer screening

The lack of physician recommendations to undergo screening tests emerged as a major barrier to screening uptake, based on the responses of more than 70% of the respondents in this study. Among those who practiced screening for CRC, the majority of the participants cited they did the test because their physician advised them to. This finding is similar to what was found in Saudi Arabia and Pakistan, where the majority of the participants reported that they would go for CRC screening if they were advised by their physician [8, 13]. This finding proves that patients often rely on the expertise and guidance of their healthcare providers, and so when physicians recommend a screening test, it reinforces the importance and benefits of the test, which can increase patient compliance. Physicians play a crucial role in patient education and raising awareness about the importance of preventive screenings. When patients are informed by their physicians about the benefits, risks, and necessity of colorectal cancer screening, they are more likely to follow through with the recommended tests. This is similar to a study in Jordan that cited the lack of physician endorsement as a barrier to screening for CRC [14]. Visiting the physician for check-ups regularly was associated with the practice of CRC screening. A similar result was found in a study in Hong Kong, which cited that visiting the physician five times or more in a year was an enabling factor in CRC screening uptake [15]. Regular check-ups involve discussions about various health-related topics, including preventive screenings. These discussions contribute to increasing individuals’ awareness and knowledge of CRC and the importance of screening. By visiting their physicians regularly, individuals receive ongoing education and updates about CRC screening guidelines, advances in screening technologies, and the potential benefits of early detection. This enhanced awareness can motivate individuals to prioritize CRC screening as part of their overall health management. A study in Lebanon further solidifies this reason in its results, where the researchers reported that participants who went for regular physician check-ups were three times more likely to have an increase in awareness about the necessity of CRC screening methods [16].

Participants in our study expressed various factors hindering them from utilizing CRC screening methods. Some of these factors include the high cost of the test, lack of knowledge, embarrassment, discomfort, lack of motivation, and the length of time taken to complete the test. These reasons are similar to what was found in a systematic review in rural USA [17]. Another study in Pakistan reported that major barriers to screening were lack of knowledge, high costs, and lack of screening facilities [13]. Considering there is a similarity with the aspect of lack of knowledge, it shows that knowledge, although high in some areas, still serves as a barrier to practicing screening for CRC. This could reflect the aspects of knowledge about cancer screening that are important. Without sufficient knowledge about colorectal cancer and its potential risks, individuals may not understand the significance of undergoing screening tests. They may underestimate the prevalence and potential severity of CRC, leading them to overlook the need for screening. Lack of knowledge about the various screening methods and options available for CRC can be a barrier to uptake. This is because people may not be aware of different screening tests like colonoscopy, fecal occult blood test (FOBT), or sigmoidoscopy, which can hinder them from making informed decisions about the screening method that best suits their circumstances and the national guidelines. Although a majority of the participants knew the various screening tests, the majority of them still reported a lack of knowledge as a barrier to the practice of CRC screening. This finding may seem contradictory, but while individuals may have some awareness of CRC screening tests, their understanding and interpretation of the information might be incomplete or inaccurate. They may be aware that screening tests exist but may not fully comprehend the importance, recommended frequency, or specific details of the procedures. As a result, they may mistakenly believe that their knowledge is insufficient, leading them to perceive a lack of knowledge as a barrier. Having health insurance was not found to be associated with the practice of CRC screening. While health insurance coverage can improve access to healthcare services, including preventive screenings, other factors can influence individuals’ utilization of CRC screening. For example, individuals may still face barriers related to awareness, knowledge, beliefs, cultural factors, or personal preferences that affect their decision to undergo screening, regardless of their insurance status. Another possibility for this finding could be that even though health insurance may cover a portion or all of the cost of CRC screening, individuals may still incur out-of-pocket expenses such as deductibles, copayments, or coinsurance. These costs can vary depending on the insurance plan and the specific screening modality chosen, and if individuals perceive the financial burden as significant, they may be less likely to undergo screening, even with insurance coverage. A systematic review reported that some studies found an association between health insurance and the practice of cancer screening, and other studies did not find any association [17].

Although the findings of this study reflect poor uptake of the CRC screening tests by adults who are eligible to get screened for the disease in the UAE, several factors may enhance this practice. These factors include being married, advanced age, being covered by health insurance, visiting the physician regularly for checkups, having a positive family history of CRC, knowing that CRC is a disease that exists, having an above-average level of knowledge about CRC as a disease, and having an above-average level of knowledge about the signs and symptoms of CRC. The most common factor reported to hinder CRC screening by participants was that they were not offered a CRC screening test by their physician. Thus, healthcare providers are encouraged to educate their patients about the importance of CRC screening and to offer them the test whenever it is possible to improve the level of uptake among eligible individuals in the UAE.

### Limitations

This study has some limitations. Firstly, the distribution of the regions based on the WHO regions is not representative of the UAE’s population structure because more than 80% of the sample is from the EMR region alone. Secondly, the frequency distribution of the participants based on the place of residence is not representative of the distribution from different emirates in the UAE. In addition, there was a lack of some demographic variables, like socioeconomic status and whether the participant is a healthcare worker, which may have an influence on the practice of CRC screening. Finally, the sample selection was not random, as no access to the sampling frame is possible.

## Conclusion

In this study, 2100 participants were recruited from all seven Emirates of the UAE, aiming to determine the practice and factors associated with CRC screening. This study indicates that CRC screening practice in the UAE is 9.1%. The CRC screening is associated with an increase in age, regular physician checkups, a positive family history of CRC, knowledge of the existence of CRC, and knowledge about CRC disease and its signs and symptoms. Although knowledge of CRC as a disease and knowledge of its signs and symptoms are above average, there is still a deficiency in screening uptake among adults in the UAE. The main factors hindering the practice of CRC screening include not being offered a screening test by the physician, fear of positive results, discomfort with the test, pain concerns, and a lack of knowledge about CRC screening.

Despite the fact that screening methods are available in the UAE, the practice is still low. Thus, more efforts are required to enhance the practice of CRC screening. From a public health perspective, education can be the key to increasing awareness about colorectal cancer and the importance of screening in the early detection of CRC cases to improve outcomes.

## Data Availability

The datasets used and/or analysed during the current study are available from the corresponding author on reasonable request.
